# Interactions Between Bacterivorous Nematodes and Bacteria Reduce N_2_O Emissions

**DOI:** 10.1002/advs.202413227

**Published:** 2025-01-31

**Authors:** Xu Xu, Xinling Wang, Ting Sun, Shanshan Liu, Menghui Dong, Yang Yue, Yi Min, Alexandre Jousset, Xian Xiao, Shuwei Liu, Stefan Geisen, Valentyna Krashevska, Qirong Shen, Stefan Scheu, Rong Li

**Affiliations:** ^1^ The Sanya Institute of the Nanjing Agricultural University Educational Ministry Engineering Center of Resource‐Saving Fertilizers Jiangsu Provincial Key Lab for Solid Organic Waste Utilization Jiangsu Collaborative Innovation Center of Solid Organic Wastes Nanjing Agricultural University Nanjing Jiangsu 210095 China; ^2^ JF Blumenbach Institute of Zoology and Anthropology University of Göttingen 37073 Göttingen Germany; ^3^ Ecology and Biodiversity Group Institute of Environmental Biology Department of Biology Utrecht University Padualaan 8 Utrecht 3584 CH The Netherlands; ^4^ Department of Agroecology Faculty of Technical Sciences Aarhus University Forsøgsvej 1 Slagelse 4200 Denmark; ^5^ School of Environmental and Safety Engineering Changzhou University Changzhou 213164 China; ^6^ Jiangsu Key Laboratory of Low Carbon Agriculture and GHGs Mitigation College of Resources and Environmental Sciences Nanjing Agricultural University Nanjing 210095 China; ^7^ Laboratory of Nematology Wageningen University Wageningen 6700 AA The Netherlands; ^8^ Senckenberg Biodiversity and Climate Research Centre Functional Environmental Genomics Senckenberganlage 25 60325 Frankfurt Germany; ^9^ Centre of Biodiversity and Sustainable Land Use 37073 Göttingen Germany

**Keywords:** nematoda, nosZ gene, selective predation, soil microbial communities, trophic interaction

## Abstract

Trophic interactions in micro‐food webs, such as those between nematodes and their bacterial prey, affect nitrogen cycling in soils, potentially changing nitrous oxide (N_2_O) production and consumption. However, how nematode‐mediated changes in soil bacterial community composition affect soil N_2_O emissions is largely unknown. Here, microcosm experiments are performed with the bacterial feeding nematode *Protorhabditis* to explore the potential of nematodes in regulating microbial communities and thereby soil N_2_O emissions. Removal of nematodes by defaunation resulted in increased N_2_O emissions, with the removal of *Protorhabditis* contributing most to this increase. Further, inoculation with *Protorhabditis* altered bacterial community composition and increased the relative abundance of *Bacillus*, and the abundance of the *nosZ* gene in soil. In vitro experiments indicated that *Protorhabditis* reinforce the reduction in N_2_O emissions by *Bacillus* due to suppressing competitors and producing bacteria growth stimulating substances such as betaine. The results indicate that interactions between nematodes and bacteria modify N_2_O emissions providing the perspective for the mitigation of greenhouse gas emissions via manipulating trophic interactions in soil.

## Graphical abstract

Multi‐methodological approaches are used, including microcosm‐based insights on potential microbiome‐nematode links and a range of follow‐up confirmative experiments to show that bacterivorous nematodes significantly increased the abundance of denitrifying bacteria, suggesting that facilitation rather than predation predominated in interactions between bacterivorous nematodes and bacteria, with these interactions changing bacterial N_2_O emission from soil.

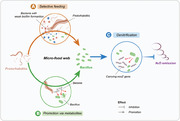

## 1 Introduction

Nitrous oxide (N_2_O) is a potent long‐lived greenhouse gas, with a global warming potential 300 times that of carbon dioxide, contributing to climate change and also ozone depletion.^[^ ^1^ ^]^ Reducing N_2_O emissions is essential to mitigate climate change impacts. N_2_O emissions come from a variety of sources, including agriculture, fossil fuel combustion, and industrial processes.^[^ ^2^, ^3^ ^]^ Agricultural soils are a major source of atmospheric N_2_O emissions, primarily driven by microbial processes such as nitrification and denitrification.^[^ ^4^ ^]^ N_2_O is produced as a byproduct of nitrification under oxic conditions, while in sub‐oxic or anoxic conditions, N_2_O emissions result from incomplete denitrification due to the inhibition of N_2_O reductase, which converts N_2_O to nitrogen.^[^ ^5^ ^]^ Importantly, N_2_O emissions represent a net balance between its formation and reduction, with microbial activities mediating both processes.

Understanding the role of microbial communities in N_2_O emissions is critical. Soil bacteria and fungi mediate denitrification processes, with the *nosZ* gene encoding N_2_O reductase playing a key role in the final step of denitrification.^[^ ^6^, ^7^ ^]^ For example, nitrogen‐fixing rhizobia equipped with denitrification genes can mitigate N_2_O emissions,^[^ ^8^ ^]^ while cooperation between arbuscular mycorrhizal fungi (AMF) and N_2_O‐reducing *Pseudomonas* species enhances N_2_O reduction by recruiting and stimulating bacterial communities.^[^ ^9^ ^]^ Notably, bacteria play a more important role in reducing N_2_O emissions than AMF due to their higher denitrification efficiency, wide ecological adaptability, and functional diversity.^[^ ^10^, ^11^ ^]^ Moreover, microbial processes are strongly influenced by soil physical and chemical factors, such as oxygen availability, carbon content, and soil moisture, which control microbial activity and the balance between nitrification and denitrification.^[^ ^4^ ^]^

Predators of bacteria and fungi such as predatory nematodes play a crucial role in regulating microbial community composition^[^ ^12^ ^]^ and their functioning.^[^ ^13^, ^14^, ^15^ ^]^ Nematodes preferentially feed on certain groups of microbes, resulting in changes in the composition and activity of microbial communities.^[^ ^16^, ^17^ ^]^ For instance, bacteria forming biofilms and producing toxins protect themselves against predators.^[^ ^18^, ^19^ ^]^ Moreover, many bacterivorous nematodes secrete signaling molecules such as ascarosides playing an important role in regulating their behavior and development, but they may also affect interactions with microorganisms,^[^ ^20^, ^21^ ^]^ thereby affecting soil nutrient cycling and plant growth.^[^ ^22^ ^]^ Recent studies also suggest that nematodes influence soil N_2_O emission.^[^ ^23^, ^24^ ^]^ However, the underlying mechanisms remain largely unexplored. Interactions between nematodes and microorganisms affecting N_2_O emissions depend on a number of factors including nematode species and microbial community composition as well as interaction type, i.e., inhibition, such as suppression of bacteria via predation, or facilitation, i.e., stimulation of bacterial abundance via nematode metabolites. *Protorhabditis* nematodes are abundant bacterivorous nematodes in soils worldwide, especially in moist soils and soils rich in organic matter. In soil microbial food webs *Protorhabditis* has been shown to promote the abundance of Gram‐positive bacteria by preying on Gram‐negative bacteria, and stimulate the overall abundance of bacteria thereby accelerating the mineralization of organic matter and the availability of nutrients such as nitrogen and phosphorus to plants.^[^ ^25^ ^]^ Understanding the interactions between nematodes, especially the widely distributed bacterivorous nematodes, and microbial communities in soil, and how they affect the activity of soil bacteria involved in N_2_O production and consumption may help in developing strategies for reducing N_2_O emissions and mitigating climate change.

We investigated whether bacterivorous nematode communities can reduce soil N_2_O emissions, and explore the mechanisms responsible for nematode‐mediated changes in N_2_O emissions via altering microbial community composition. We hypothesized that 1) the presence of bacterivorous nematodes stimulates the abundance of bacteria and changes bacterial communities, and 2) stimulation of bacteria by nematodes is associated by increased abundance of *nosZ* genes in soil resulting in reduced N_2_O emission. We first identified the major players and pathways by investigating soil microbial community composition and functional genes (*nosZ* I, *nosZ* II, *nirS*, and *nirK*) involved in denitrification. Then, according to our high‐throughput sequencing analysis, we isolated the most responsive bacteria (*Bacillus* and *Neobacillus*) to the presence of nematodes and tested the growth response of bacterial isolates to the bacterivorous nematode *Protorhabditis*. Subsequently, the target strains were reinoculated into nematode‐free soil to validate the results of the in vitro cultures. Finally, the combined effect of *Bacillus* and *Protorhabditis* in soil was investigated. Overall, the study aims to provide deeper insight into the potential of bacterivorous nematodes in regulating microbial community composition and thereby reducing N_2_O emissions from soils.

## 2 Experimental Section

### 2.1 Microcosm Experiments

For the microcosm experiments, arable soil was taken from a long‐term field experiment of the Nanjing Institute of Vegetable Science in the vicinity of Nanjing located in the subtropical monsoon climate region in China (31°43' N, 118°46′ E).^[^ ^26^ ^]^ The soil comprised hydroponic artificial soil developed from loess parent material with clay loam texture, more details on physical and chemical properties are given in the supplementary materials (Table S1, Supporting Information). During the growing season (June – September) mineral fertilizers were added to experimental plots at an amount of 120 kg ha^−1^ nitrogen, 180 kg ha^−1^ phosphorus, and 120 kg ha^−1^ potassium. The soil was collected in autumn (October), passed through 4 mm mesh to remove roots and stones, and then divided into two parts. One part was sterilized by 75 kGy gamma rays at Nanjing Xiyue Technology Co., Ltd, Nanjing, China. The other part was used to prepare soil suspensions at a volume ratio of 1:20 soil and sterile water, and passed through a 20 µm mesh to obtain nematode‐free suspensions.^[^ ^27^ ^]^ The absence of nematodes including eggs in the suspensions was checked by microscopic observation. The nematode‐free suspension was added to sterile soil at a volume ratio of 1:10 to produce nematode‐free soils. Then, the soil was stored in sealed plastic culture bottles at 25 °C for ≈1 month to stabilize microbial communities^[^ ^28^ ^]^ (Figure S1, Supporting Information). Prior to being used, soil moisture was adjusted to 60% maximum water‐holding capacity by adding distilled water. Nematode‐free soil was used as control in all the experiments.

#### 2.1.1 Microcosm Experiment 1

**Figure 1:**
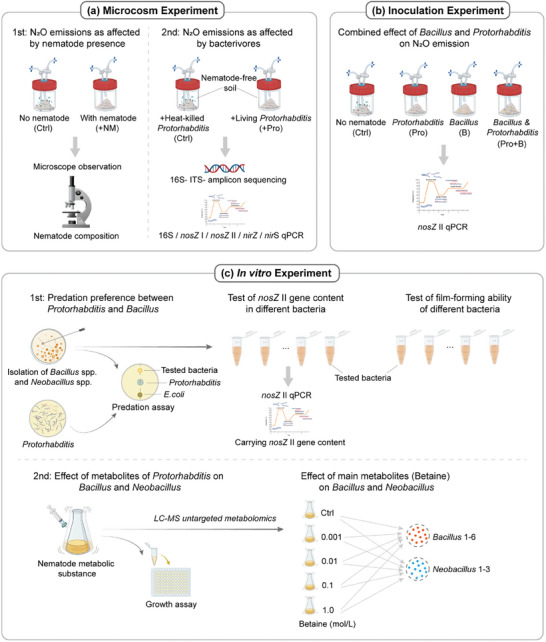
Flow chart of the experiments. The study comprised five experiments: two microcosm experiments, an inoculation experiment, and two in vitro experiments. a) The microcosm experiments 1 and 2 tested the effects of nematodes on N_2_O emission and the composition of the microbial community; b) the inoculation experiment assessed the individual and combined effects of *Bacillus* and *Protorhabditis* on N_2_O emission and *nosZ* II gene abundance; c) the two in vitro experiment assessed predation preference (1st experiment) and effects of nematode metabolites (2nd experiment) of the soil bacterivorous nematode *Protorhabditis* on the investigated bacterial strains.

Microcosm experiment 1 was conducted to examine if nematode inoculation reduces N_2_O emissions. Nematode suspension was collected from 100 g field soil using the shallow dish method.^[^ ^29^ ^]^ Then, the nematode suspension was added to pots containing 100 g soil, establishing two treatments, i.e., nematode‐free soil with the addition of sterile water only (Ctrl) and nematode‐free soil inoculated with nematode suspension (+NM), each with six replicates (**Figure** **1**). The microcosms were incubated at 25 °C, with soil moisture maintained at 25% (w/w). N₂O concentrations were measured every two days until stabilization.^[^ ^30^ ^]^

Before each sampling, standard air was used to flush the microcosms at a rate of 300 mL min^−1^ for 10 min. Then, the culture bottles were sealed and incubated at 25 °C temperature for 4 h. Before (0 h) and after the incubation period (4 h) 20 mL of the headspace of the microcosms was collected through a gas sampling needle and injected into a 20 mL vacuum bottle. N_2_O concentrations were measured by a modified gas chromatograph (Agilent 7890A, Agilent Technologies, USA) equipped with a flame ionization detector (FID) and an electron capture detector (ECD). The N_2_O fluxes were determined by a nonlinear fitting approach. The emission rate of N_2_O (fN_2_O, µg kg^−1^ h^−1^) was calculated as:

(1)

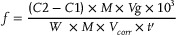

where *C*1 and *C*2 are the N_2_O gas concentrations at 0 and 4 h a of incubation (µL L^−1^), *M* is the molar weight of N_2_O‐N (g mol^−1^), Vg is the headspace volume of the culture bottle (m^3^), *W* is the weight of the soil in the culture bottle (kg), *t*’ is the interval time (4 h), and *V*_corr_ is the molecular volume after temperature correction (m^3^ mol^−1^), which was calculated as:

(2) $$Vcorr=0.02241\times \frac{273.15+T}{273.15}$$

where *T* is the temperature of the culture, 0.02241 is the molar volume (m^3^) of an ideal gas at 1 atm and 273.15 K.

To explore the composition of soil nematode communities, we combined a modified Baermann funnel technique and gradient centrifugation for nematode extraction.^[^ ^29^ ^]^ Specifically, soil samples were first placed in shallow water‐filled plastic dishes, allowing nematodes to migrate out of the soil into the water over a period of 48 h at room temperature. This migration technique, based on the Baermann funnel principle, facilitates the collection of actively moving nematodes. Subsequently, the collected nematodes were concentrated and further purified using a sucrose density gradient centrifugation method to separate nematodes from soil debris and other particulates. Nematodes were carefully washed from the sieve into plastic dishes and then counted under a dissecting microscope (Nikon ECLIPSE Ts2) at 40× magnification. From each microcosm six samples were inspected. In addition, ≈100 nematode individuals were randomly picked from the plastic dishes and transferred to nematode growth medium (NGM; 2.5 g peptone, 3.0 g sodium chloride, 0.111 g calcium chloride, 0.120 g magnesium sulfate, 0.005 g cholesterol, 3.4 g monobasic tripotassium phosphate, and 17.0 g agar) inoculated with *Escherichia coli* as a food source for the nematodes. Based on differences in morphological characters, individual nematodes were picked and cultivated on NGM plates to obtain pure cultures. One frequently isolated strain with high activity and reproduction was identified as belonging to the genus *Protorhabditis*. This identification was initially based on microscopic examination and later confirmed through sequencing the total DNA of 50 nematodes, followed by comparison with NCBI database records^[^ ^31^, ^32^ ^]^ and this isolate was further cultivated on NGM plates at 22 °C.

#### 2.1.2 Microcosm Experiment 2

Microcosm experiment 2 was conducted to examine the potential of *Protorhabditis* to reduce N_2_O emissions. Based on the nematode community isolated and detected in microcosm experiment 1, *Protorhabditis* as dominant genus, with its abundance significantly negatively correlating with N_2_O emissions, was selected for this microcosm experiment. Two treatments were established: 1) sealed jars containing 100 g sterilized soil (60% maximum water‐holding capacity) with 20–30 living *Protorhabditis* individuals per gram soil, and 2) a respective number of heat‐killed *Protorhabditis* individuals (control). For each treatment, six replicates were set up, and microcosms were kept at room temperature. N₂O concentrations were measured every two days, with a total of seven recordings taken until the levels stabilized. (Figure 1).

By the end of the experiment, soil samples were taken as described previously.^[^ ^33^ ^]^ Soil DNA was extracted from 0.5 g soil using the DNeasy Power max Soil Kit (Qiagen, Germany) according to the manufacturer's instructions. A NanoDrop ND2000 (Thermo Scientific, Waltham, MA, USA) spectrophotometer was used to measure the genomic DNA concentration and purity to meet the quantitative real‐time PCR amplification (qPCR) assay and Illumina sequencing requirements. For bacteria, the V4 hypervariable region of the 16S rRNA gene was amplified using the primer pairs 515F (5′‐GTGCCAGCMGCCGCGGTAA‐3′) and 806R (5′‐GGACTACHVGGGTWTCTAAT‐3′);^[^ ^34^ ^]^ for fungi, ITS genes were amplified using the paired primers ITS1F (5′‐CTTGGTCATTTAGAGGAAGTAA‐3′) and ITS2 (5′‐GCTGCGTTCTTCATCGATGC‐3′).^[^ ^35^ ^]^ Detailed information regarding the PCR program and barcode strategy for each sample is provided in the Supplementary Material (Table S2 and Figure S2, Supporting Information). The amplification product was sequenced on an Illumina MiSeq platform at Personal Biotechnology Co., Ltd. (Shanghai, China). The abundance of bacteria was quantified by qPCR using the above primers.^[^ ^34^ ^]^ The abundance of the *nosZ* I gene was quantified using the primers *nosZ*‐2F (5′‐ CGCRACGGCAASAAGGTSMSSGT ‐3′) and *nosZ*‐2R (5′‐ CAKRTGCAKSGCRTGGCAGAA‐3′),^[^ ^36^ ^]^ and the *nosZ* II gene was quantified using the primers *nosZ*‐II‐F (5′‐ CTIGGICCIYTKCAYAC – 3′) and *nosZ*‐II‐R (5′‐ GCIGARCARAAITCBGTRC – 3′).^[^ ^37^ ^]^ The abundance of the *nirS* gene was quantified using the primers cd3aF (5′‐GTSAACGTSAAGGARACSGG‐3′) and R3 cd (5′‐GASTTCGGRTGSGTCTTGA‐3′).^[^ ^38^ ^]^ The abundance of the *nirK* gene was quantified using the primers F1aCu (5′‐ATCATGGTSCTGCCGCG‐3′) and R3Cu (5′‐GCCTCGATCAGRTTGTGGTT‐3′).^[^ ^39^ ^]^ qPCR analyses were performed with a qTOWER 2.2 system (Analytik Jena, Jena, Germany) using SYBR green I fluorescent dye detection in 20 µL volumes, which contained 2 µL of template, 10 µL of SYBR Premix Ex Taq (TaKaRa Bio Inc., Japan), and 0.4 µL of both forward and reverse primers (10 µm each) in 96‐well plates.^[^ ^40^ ^]^ All qPCRs were performed using the standard temperature profile at Personal Biotechnology Co., Ltd. (Shanghai, China). The qPCR efficiencies of each gene in denitrification are given in the supplementary materials (Table S3, Supporting Information). Six replicates were analyzed per sample and the results were expressed as log10 values (target copy number per gram soil).

According to our high‐throughput sequencing analysis and 16S qPCR, increased rather than decreased bacterial abundance was the main factor affecting N_2_O emissions. Among all bacteria, *Bacillus* and *Neobacillus* occupied a dominant position and correlated closely with N_2_O emissions. Therefore, we isolated *Bacillus* and *Neobacillus* strains from the treatment with *Protorhabditis*. In brief, 5 g soil was suspended in 250 mL Erlenmeyer flasks containing 45 mL sterile distilled water. After stirring at 180 rpm for 40 min, the soil suspension was placed at 80 °C in a water bath for 30 min. Then, serial dilutions were prepared and spread onto plates containing nutrient agar (NA) medium amended with cycloheximide (100 µg mL^−1^). The plates were incubated at 37 °C for 48 h. *Bacillus* and *Neobacillus* colonies were preliminarily identified based on morphology and Gram staining. *Bacillus* and *Neobacillus* colonies were then purified using NA medium amended with cycloheximide (100 µg ml^−1^) and identified based on the full‐length 16S rRNA gene sequence as described previously.^[^ ^41^ ^]^ Specifically, genomic DNA was extracted, and the 16S rRNA gene was amplified using polymerase chain reaction (PCR) using the following primers: 5′‐AGAGTTTGATCCTGGCTCAG‐3′ and 5′‐TAC GGTTACCTTGTTACGACTT‐30′. The 16S rRNA gene sequence of all strains was sequenced and analyzed using BLAST searches. The phylogenetic tree was built with the neighbor‐joining method.^[^ ^42^ ^]^

### 2.2 Inoculation Experiment

An inoculation experiment was established to examine the effects of *Bacillus* and the combination of *Bacillus* and *Protorhabditis* on the abundance of the *nosZ* gene and N_2_O emission. The experiment was set up in a similar way as the microcosm experiments. Based on the results of high‐throughput sequencing analysis and our in vitro experiments (see below), six bacterial isolates (*Bacillus* 1–6) and the bacterivorous nematode *Protorhabditis* were selected to examine effects of *Bacillus* and the combination of *Bacillus* and *Protorhabditis* on the abundance of the *nosZ* gene and N_2_O emission. The experiment was set up in a similar way as the microcosm experiments. A total of 2000 *Protorhabditis* individuals and 5 mL of each bacterial solution (10^6^ cfu mL^−1^) were introduced into 200 g soil per pot; 14 treatments were set up: 1) nematode‐free soil (Ctrl) established by adding 5 mL of sterile water, 2) nematode‐free soil inoculated with *Protorhabditis* (Pro), 3–8) nematode‐free soil inoculated with each *Bacillus* strain (B1‐B6), and 9–14) nematode‐free soil inoculated with each *Bacillus* strain and *Protorhabditis*. After incubation at 25 °C for 2 weeks N_2_O concentration in the headspace of each microcosm sealed for 4 h was measured as well as the abundances of the *nosZ* gene. Moreover, the abundances of *Bacillus* and total bacteria were measured for successful colonization and dominance of the inoculated species ensured. Soil moisture was adjusted to 60% maximum water‐holding capacity by adding distilled water. Three replicates were set up for each treatment.

### 2.3 In Vitro Experiments

In the two in vitro experiments, the nematode suspension was obtained by rinsing *Protorhabditis* from culture plates into 50 mL centrifuge tubes using sterile water. The suspension then was allowed to stand for 30 min. Thereafter, the supernatant was discarded, the nematodes were rinsed with sterile water and centrifuged at 3000 rpm for 1 min. This procedure was repeated three times to reduce the number of *E. coli* in the suspension.^[^ ^43^ ^]^ According to sequencing analysis results, *Bacillus* and *Neobacillus* genes strongly responded to the presence of bacterivorous nematodes, and correlated significantly with N_2_O emissions (*Bacillus* negative, *Neobacillus* positive). To obtain solutions of the six *Bacillus* strains and three *Neobacillus* strains, *Bacillus* and *Neobacillus* colonies were added to 5 mL nutrient broth medium and cultured at 250 rpm and 30 °C for 48 h. Then, the suspension was centrifuged at 5000 rpm for 10 min, the supernatant was discarded, and sterile water was added.

#### 2.3.1 In Vitro Experiment 1

Predation preference of *Protorhabditis* for the six *Bacillus* strains and three *Neobacillus* strains were tested using a plate assay. The concentration of the nematode suspension was adjusted to ≈10000 *Protorhabditis* individuals mL^−1^. First, 2.5 µL of *Bacillus* or *Neobacillus* suspension (at a concentration of 10^6^ cfu mL^−1^) and 2.5 µL of *E. coli* suspensions (at a concentration of 10^6^ cfu mL^−1^, used as control treatment) were inoculated on opposite sides of an NGM plate and incubated at 30 °C. When the colonies reached a diameter of 0.5 cm, the center of the NGM plate was inoculated with 5 µL nematode suspension containing ≈100 *Protorhabditis* individuals. A total of nine treatments was set up: 1–7) Inoculated with each *Bacillus* strains 1–7 and *E*. *coli*, 8,9) inoculated with each *Neobacillus* strain 1 or 2 and *E*. *coli*. For each treatment, six replicates were set up. After 24 h, predation of *Protorhabditis* on *Bacillus* and *Neobacillus* was inspected under the stereomicroscope. Predation preference (P) of *Protorhabditis* was calculated as:

(3) $$P\;=\frac{P_{B}-P_{E}}{P_{B}+P_{E}}\;\times \;100\%$$

with *P*_*B*_ the number of *Protorhabditis* consuming test strains (*Bacillus* or *Neobacillus*) and *P*_*E*_ the number of *Protorhabditis* consuming *E*. *coli*.

Moreover, the total bacterial DNA of each isolate was extracted from 0.5 mL culture suspension (OD_600_ = 0.5) with a FastPure Bacteria DNA Isolation Mini Kit (Vazyme Biotech, Nanjing, China). The bacterial primers *nosZ*‐1527F (5′‐CGCTGTTCHTCGACAGYCA‐3′) and *nosZ*‐1773R (5′‐ATRTCGATCARCTGBTCGTT‐3′) were used for *nosZ* gene amplification to examine whether the *Bacillus* isolates possessed the *nosZ* gene as previously described.^[^ ^9^, ^44^ ^]^ The biofilm formation of *Bacillus* and *Neobacillus* isolates were tested as previously described.^[^ ^40^ ^]^ In brief, exponential phase cultures of *Bacillus* and *Neobacillus* isolates were adjusted to an optical density at 600 nm (OD_600_) of 0.15 in nutrient broth medium and then inoculated on Nunc‐TSP plates. The inoculum volumes were 160 µL for NB and 40 µL of bacterial suspensions. After incubation at 30 °C for 72 h, biofilm formation was quantified by a modified crystal violet (CV) assay. Six replicates were set up for each treatment.

#### 2.3.2 In Vitro Experiment 2

To explore effects of *Protorhabditis* metabolites on the investigated *Bacillus* strains, we established a coculture system. First, the density of *Protorhabditis* cultivated at 250 rpm and 22 °C was adjusted to 15000 ind. mL^−1^ using sterile water. Then, 10 mL *Protorhabditis* suspensions were centrifuged at 8000 rpm at 4 °C for 15 min, filtered through 0.22 µm membrane, freeze‐dried, and stored at −80 °C.^[^ ^45^, ^46^ ^]^ Half of the metabolite solution was used for the coculture system, the other half was frozen in liquid nitrogen and sent to Personalbio Technology Co., Ltd, Nanjing, China (http://www.personalbio.cn/) for analyzing chemical compounds. The coculture system was set up in 96‐well plates to which 5 µL bacterial suspension, 10 µL nematode metabolite suspension and 185 µL nutrient broth medium was added. Control systems received 5 µL bacterial suspension, 10 µL sterile water and 185 µL nutrient broth medium.^[^ ^33^ ^]^ Each treatment was replicated six times. The vials were incubated at 30 °C for 3 days. The optical density (OD_600_) of the suspension was measured twice a day using SpectraMax M5 (Sunnyvale, CA, USA).

To explore the effects of one major compound in the metabolite suspension (betaine) on the growth of the six *Bacillus* strains, we set up a third coculture system. This system was set up in a similar way as above but with 5 µL bacterial suspensions, 20 µL betaine solution, and 175 µL nutrient broth medium. The control treatment received 20 µL sterile water instead of betaine solution. Four concentrations of betaine were tested, 0.001, 0.01, 0.10, and 1.00 mol L^−1^. Six replicates were set up for each treatment. The vials were incubated as described above. The growth rates of the isolates were measured though optical density (OD_600_) of the suspension as describe above.

### 2.4 Statistical Analyses

Bacterial and fungal raw sequences were split. Adaptors and primer sequences were trimmed using cutadapt (https://github.com/marcelm/cutadapt).^[^ ^47^ ^]^ Subsequently, the trimmed bacterial and fungal sequences were processed using the UPARSE pipeline.^[^ ^48^ ^]^ Bacterial and fungal sequences with a quality score lower than 0.5 or shorter than 200 bp were removed. After discarding singletons, bacterial and fungal sequences were categorized into OTUs at 97% similarity.^[^ ^49^ ^]^ The RDP database version 11.5 (http://rdp.cme.msu.edu/) and UNITE database version 9.0 (https://unite.ut.ee/) were used to identify the taxonomic affiliation of bacterial and fungal OTUs, respectively.^[^ ^42^ ^]^ For each treatment, the average value and standard deviation was calculated from six replicates.

For analyzing nematode metabolite data, the raw data were converted to mzXML format by MSConvert in ProteoWizard software package (v3.0.8789)^[^ ^50^ ^]^ and processed using XCMS^[^ ^51^ ^]^ for feature detection, retention time correction, and alignment. Nematode metabolites were identified by accuracy mass (< 30 ppm) and MS/MS data matching with HMDB (http://www.hmdb.ca),^[^ ^52^ ^]^ massbank (http://www.massbank.jp/),^[^ ^53^ ^]^ LipidMaps (http://www.lipidmaps.org),^[^ ^54^ ^]^ mzcloud (https://www.mzcloud.org)^[^ ^55^ ^]^ and KEGG (http://www.genome.jp/kegg/).^[^ ^56^ ^]^ Robust LOESS signal correction (QC‐RLSC)^[^ ^57^ ^]^ was applied for data normalization to correct for systematic bias. After normalization, only peaks with relative standard deviations < 30% in quality control were kept to ensure correct metabolite identification.

The α‐diversity of bacterial and fungal communities was estimated using the nonparametric Shannon index.^[^ ^58^ ^]^ Principal coordinate analysis (PCoA) based on Bray–Curtis distance metrics was used to explore differences in bacterial and fungal community composition.^[^ ^59^ ^]^ Two‐way ANOVA was performed to assess the effects of nematode addition on the diversity (Shannon index) of bacteria and fungi using SPSS v20.0 (SPSS Inc., Chicago, USA).^[^ ^47^ ^]^ Permutational multivariate analysis of variance (PERMANOVA)^[^ ^60^ ^]^ was used to assess the effect of *Protorhabditis* on the community composition of microbial groups using the adonis function with 999 permutations in the “vegan” package in R (version 4.0.1).^[^ ^61^ ^]^ Spearman correlations were calculated between bacterial relative abundance and N_2_O emission using the “ggcor” package in R. Linear discriminant analysis (LDA) effect size (LEfSe) was calculated to identify significant differences in relative abundant bacterial and fungal taxa between *Protorhabditis* and control treatments.^[^ ^62^ ^]^ The threshold for the logarithmic LDA score for identifying significant differences was 2.5. LEfSe analysis was performed using the Huttenhower lab Galaxy server (http:// huttenhower.sph.harvard.edu/galaxy).^[^ ^62^ ^]^ Structural equation modeling (SEM) was conducted using IBM SPSS Amos 26 (Chicago, IL: Amos Development Corporation).^[^ ^63^ ^]^ It was used to evaluate direct and indirect effects of nematodes and bacterial communities on N_2_O emission. In the model, the treatments (control, +Pro) were used as categorical variables.^[^ ^64^ ^]^ We also calculated the standardized total effects (STEs) of *Bacillus* OTU 834, soil properties (NH_4_^+^‐N and NO_3_^−^‐N), and *nosZ* effect on the N_2_O emission to aid interpretation of SEM results.

## 3 Results

### 3.1 Microcosm Experiment

**Figure 2:**
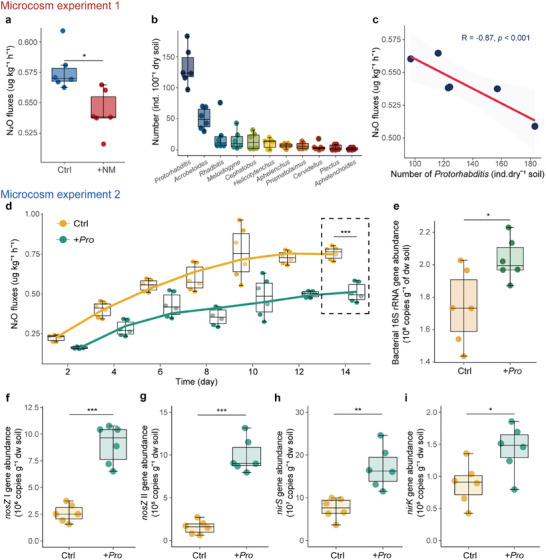
N_2_O emission from soil in the absence and presence of nematodes. Microcosm experiment 1: a) N_2_O flux from soil in absence (Ctrl) and presence of nematodes (+NM) after 2 weeks; asterisks indicate significant difference between Ctrl and +NM treatments, unpaired Student's *t*‐test (^*^, *p* < 0.05). b) Number of nematodes of different genera in the treatment with nematodes (+NM). c) Relationship between the number of *Protorhabditis* and N_2_O flux. Microcosm experiment 2: d) Temporal dynamics of N_2_O fluxes in the control (Ctrl) and the treatment with *Protorhabditis* (+Pro) (*n* = 6); data points are slightly shifted to improve visibility; e) Abundances of the bacterial 16S rRNA in the control (Ctrl) and the treatment with *Protorhabditis* (+Pro). f–i) Abundances of the denitrification genes *nosZ* I, *nosZ* II, *nirS*, and *nirK* (copies g^−1^ dw soil) in the absence and presence of *Protorhabditis*. Asterisks indicate significant difference between the Ctrl and +Pro treatments, unpaired Student's *t*‐test (^*^, *p* < 0.05; ^**^, *p* < 0.01; ^***^, *p* < 0.001).

In microcosm experiment 1, N_2_O emission in the +NM treatment was 6.1% lower than in the Ctrl treatment (**Figure** **2**a). In the +NM treatment *Protorhabditis* was the dominant nematode genus (Figure 2b) and its abundance negatively correlated with N_2_O fluxes (R = −0.87, *p* < 0.001; Figure 2c).

In microcosm experiment 2, N_2_O emissions increased with the inoculation with *Protorhabditis* reducing N_2_O emission throughout the experiment (Figure 2d; *p* < 0.001, unpaired Student's *t*‐test). At the end of the experiment, the NH_4_^+^‐N concentration in soil with nematodes was slightly but significantly higher (+2.6%) than that in the control (*p* < 0.001; Figure S3, Supporting Information), whereas the NO_3_^−^‐N concentration was slightly but significantly reduced (−0.57%, *p* < 0.01; Figure S4, Supporting Information).

Further, the abundance of 16S rRNA genes was increased in the nematode treatment (*p* < 0.05, Figure 2e). Moreover, the inoculation with *Protorhabditis* significantly increased the abundances of denitrification genes (*nosZ* I, *nosZ* II, *nirK*, and *nirS*) (Figure 2e–i) and decreased the (*nirK* + *nirS*) / *nosZ* ratio, while it did not significantly affect the *nosZ I* / *nosZ II* ratio (Figure S5, Supporting Information). Among these genes and genes ratio, the abundance of *nosZ* II gene experienced the most significant change, with the presence of *Protorhabditis* nematodes resulting in a 5.8‐fold increase in abundance in soils compared to the control and positively correlated with the abundance of bacteria in soils (R = 0.64, *p* = 0.033; Figure S6, Supporting Information). Multiple linear regression analysis indicated that the abundance of the *nosZ* II gene is the most important and significant factor in reducing N₂O emissions (*p* < 0.001, Figure S7, Supporting Information).

**Figure 3:**
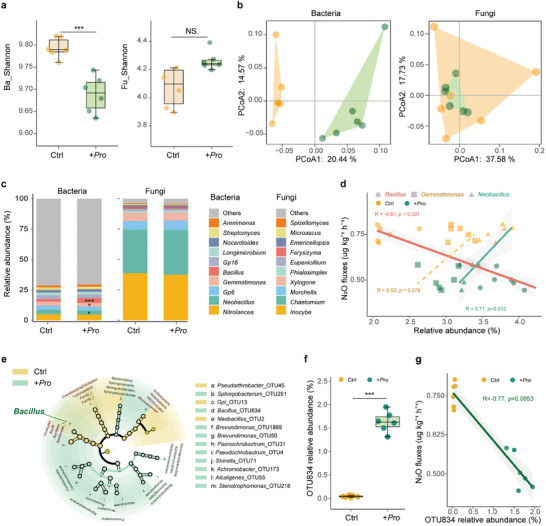
Bacterial and fungal community structure, diversity, and composition in microcosm experiment 2. a) Diversity of soil bacteria and fungi in the control (Ctrl) and the treatment with *Protorhabditis* (+Pro) (*n* = 6); asterisks indicate significant differences, unpaired Student's *t* tested (^*^, *p* < 0.05; ^**^, *p* < 0.01; ^***^, *p* < 0.001). b) Principal co‐ordinates analysis of soil bacterial and fungal OTUs; orange, control, green, +Pro. c) Relative abundance of major taxonomic groups of bacteria in the control and +Pro treatment at genus level. d) Spearman correlations between the relative abundance of different bacterial genera (*Bacillus*, *Gemmatimonas*, and *Neobacillus*) and N_2_O emission. e) Linear discriminant analysis effect size (LEfSe) identifying bacterial taxa significantly differing between the control and *+*Pro treatment; colored shading and trends of the significantly different taxa; only taxa meeting a linear discriminant analysis (LDA) significance threshold of > 2.5 are shown. f) Relative abundance of *Bacillus* OTU 834 in the control and *+*Pro treatment; asterisks indicate significant difference, unpaired Student's *t*‐test (^***^, *p* < 0.001). g) Correlation between N_2_O fluxes and the relative abundance of *Bacillus* OTU 834.

In contrast to bacterial abundance, bacterial OTU diversity was significantly reduced by the inoculation with *Protorhabditis* as indicated by the Shannon index (*p *< 0.001; **Figure** **3**a). Further, the inoculation with *Protorhabditis* significantly altered the composition of the bacterial but not the fungal community (Figure 3b). Differences in the genera composition of bacterial community were mainly due to *Bacillus*, *Gemmatimonas*, and *Neobacillus*, with the differences in *Bacillus* being most pronounced (*p* < 0.001; Figure 3c). Among them, *Bacillus* is increased (+76.2%) whereas that of *Gemmatimonas* and *Neobacillus* is decreased (−7.6% and −8.9%).

Linear discriminant analysis indicated that *Bacillus* OTU was most enriched by the inoculation with *Protorhabditis* (Figure 3e; Table S4, Supporting Information). Differences between the control and the nematode treatment were most pronounced in *Bacillus* OTU834 (Figure 3f), with its relative abundance correlating negatively with N_2_O fluxes (R = −0.77, *p* = 0.0053; Figure 3g).

**Figure 4:**
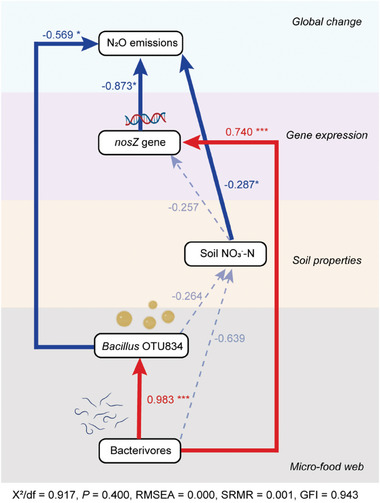
Structural equation model (SEM) on pathways by which *Protorhabditis* affects N_2_O emissions. Arrow width is proportional to the strength of the relationship. Red line, positive correlation; blue line, negative correlation. Numbers alongside the lines represent the standardized regression weights of the model. χ^2^, Chi‐square; df, degrees of freedom; GFI, goodness‐of‐fit index; CFI, comparative fit index; SRMR, standardized root mean square residual; RMSEA, root‐mean squared error of approximation are the goodness‐of‐fit statistics.

As indicated by SEM, *Protorhabditis* strongly positively affected *Bacillus* OTU 834 abundance and the abundance of *nosZ* II, which correlated negatively with N_2_O emissions. (**Figure** **4**). Of the soil properties, concentrations of NO_3_^−^‐N in soil decreased the N_2_O emission.

### 3.2 Inoculation Experiment

**Figure 5:**
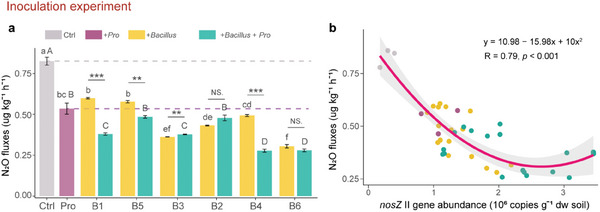
N_2_O emission suppression capability and the linkage with soil *nosZ* II gene abundance in the inoculation experiment. a) N_2_O emission in the absence (Ctrl) or presence of *Bacillus* strains (+*Bacillus*), *Protorhabditis* (+*Pro*), and the combination of both (+*Bacillus*+*Pro*). Difference of *Bacillus* and *Bacillus* + *Protorhabditis* according to unpaired Student's *t*‐test, asterisks indicate significant differences (^*^, *p* < 0.05; ^**^, *p* < 0.01; ^***^, *p* < 0.001). Different lowercase or uppercase letters indicate significant differences among treatments according to Tukey's HSD test. b) Correlation between N_2_O emission and *nosZ* II gene abundance. The “lm” function was used to fit the polynomial regression model; least squares method was used to estimate the coefficients in the model.

A total of 34 isolates taxonomically affiliated with *Bacillus* were obtained from microcosm experiment 2. Based on 16S rRNA genes these isolates comprised six bacterial strains (B1, B2, B3, B4, B5, and B6) aligned with *Bacillus* species, with isolate B6 sharing 98% sequence identity with *Bacillus* OTU 834 (Figure S8, Supporting Information). We found that the relative abundances of *Bacillus* in soils inoculated with *Bacillus*, and *Bacillus* and *Protorhabditis* nematodes were significantly increased compared to the control. The relative abundance of *Bacillus* in soil inoculated with *Bacillus*, *Protorhabditis* nematodes, and *Bacillus* and *Protorhabditis* nematodes significantly exceeded that in the control by 17.8%, 11.3%, and 9.0%, respectively (Figure S9, Supporting Information). Inoculation with these strains reduced N_2_O emissions significantly compared to nematode‐free soil (**Figure** **5**a). The inhibition was most pronounced in B6 (−63.1%) and declined in the following order: B3 (−56.1%) > B2 (−47.7%) > B4 (−40.3%) > B5 (−30.1%) > B1 (−27.4%). The additional inoculation with *Protorhabditis* further reduced N_2_O emissions in the treatments with *Bacillus* strains B1, B4, and B5 (−36.6%, −43.7%, and −16.0%, respectively) compared to the treatments only inoculated with the respective *Bacillus* strains. Further, inoculation with each of the *Bacillus* strains and the combination of *Bacillus* and *Protorhabditis* increased the abundance of the *nosZ* II gene compared to sterile control soil. Based on quadratic fitting *nosZ* II abundance significantly negatively correlated with soil N_2_O emissions (Figure 5b).

### 3.3 In Vitro Experiment

**Figure 6:**
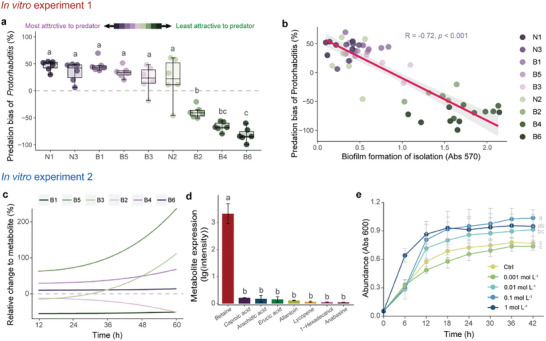
Response of *Bacillus* strains (B1‐B6) and *Neobacillus* strains (N1‐N3) to predation by *Protorhabditis* as well as exudates of *Protorhabditis* in the two in vitro experiments. In vitro experiment 1: a) Predation preference of *Protorhabditis* for *Bacillus* strains compared to *Escherichia coli*. b) Inhibition effects of *Bacillus* on soil N_2_O emissions. In vitro experiment 2: c) Relative changes (OD_600_) of the tested *Bacillus* strains to nematode metabolites compared to the control treatment; relative changes > 0 indicate increased abundance. d) The top eight most abundant nematode metabolites. Different lowercase letters indicate significant differences among treatments according to Tukey's HSD test. e) Growth curve of *Bacillus* strain B6 at different concentrations of Betaine (0.001, 0.01, 0.10, and 1.00 mol L^−1^).

In in vitro experiment 1, predation preference of *Protorhabditis* for strains N1, N3, B1, B5, B3, and N2 was significantly higher than for strains B2, B4, and B6 (**Figure** **6**a). The results of the independent samples *t*‐test indicated that the *Protorhabditis* preferred to prey on *Bacillus* rather than *Neobacillu*s (Figure S10, Supporting Information). As indicated by Spearman correlation analysis, predation bias of *Protorhabditis* against *Bacillus* and *Neobacillus* correlated significantly with the ability of bacterial biofilm formation (R = −0.72, *p* < 0.001; Figure 6b). Moreover, qPCR showed that although the abundance of *nosZ* gene was highest in *Bacillus* strains B6 and B4 whereas it was almost absent in strains in B1 and B5 (Table S5, Supporting Information), and Spearman correlation analysis showed that although there was a negative correlation between the abundance of bacteria carrying the *nosZ* II gene and the predation preference of *Protorhabditis*, the relationship was not statistically significant (R = −0.57, *p* = 0.12; Figure S10, Supporting Information).

In addition, as shown in our in vitro experiment 2, the growth of *Bacillus* strain B5 was most strongly stimulated by nematode metabolites followed by strains B3, B4, and B6, whereas the growth of strains B1 and B2 was inhibited weakly (Figure 6c). The growth of *Neobacillus* was only marginally affected by nematode metabolites (Figure S12, Supporting Information). Among the nematode metabolites identified, betaine accounted for the by far largest proportion (Figure 6d). As indicated by our growth assay, higher betaine concentrations (0.01, 0.1, and 1.0 mol L^−1^) promoted the growth of strain B6 (18.7%, 34.6%, and 23.2%, respectively), but at low concentration (0.001 mol L^−1^), there was no significant difference compared to the control (Figure 6e). High concentration of betaine (1.0 mol L^−1^) promoted the growth of both *Bacillus* and *Neobacillus* to a similar extent, whereas low concentration (0.001 mol L^−1^) had no significant effect on the growth of *Bacillus* (Figure S13, Supporting Information).

## 4 Discussion

We demonstrated that predator‒prey interactions in soil micro‐food webs change the functioning of the soil microbiome and reduce soil N_2_O emission, overall supporting our hypotheses that bacterivorous nematodes influence denitrification processes by altering bacterial communities. More specifically, we showed that soil nematodes reduce N_2_O emissions, which supports earlier findings indicating that soil faunal may mediate soil N_2_O emission.^[^ ^6^, ^65^ ^]^ Notably, we identified that the bacterivorous nematode *Protorhabditis* played an important role in driving changes in N_2_O emissions and identified changes in bacterial community composition as likely mechanism. Bacterivorous nematodes are the most abundant trophic group of nematodes, typically representing half the number of total soil nematodes.^[^ ^66^ ^]^ By feeding on microorganisms they have been shown to stimulate nutrient mineralization, in particular nitrogen and phosphorus.^[^ ^43^ ^]^ Correspondingly, we also found that *Protorhabditis* inoculation increased NH_4_^+^ concentrations in soil. NH_4_^+^ serves as the substrate for nitrification and remained at high concentration, indicating that the nitrification process was inhibited. By contrast, the concentration of NO_3_^−^ was reduced, and combed with the reduction in N_2_O emissions and increased expression of *nosZ* II encoding N_2_O reductase, we assume that NO_3_^−^ was rapidly converted during the first stage of the denitrification process, with high expression of *nosZ* II resulting in more complete denitrification. These significant changes in N_2_O emissions were associated with strong changes in the abundance of the *nosZ* gene as major gene driving N_2_O emissions. Further, inoculation with *Protorhabditis* significantly increased (rather than decreased) the abundance of bacteria as indicated by bacterial 16S rRNA gene abundance, suggesting that facilitation rather than reduction predominated in interactions between *Protorhabditis* and bacteria, with these interactions changing bacterial N_2_O emission from soil.

### 4.1 Nematode Effects on Bacterial Communities

Inoculation with *Protorhabditis* changed bacterial community composition, which supports results of former studies that, while bacterivorous nematodes and bacteria interact antagonistically, they also engage in beneficial relationships by impacting bacterial resource supply.^[^ ^67^, ^68^ ^]^ Hence, bacterivorous nematodes may differentially impact bacterial species, as supported by our study. In fact, bacterivore nematodes strongly increased the abundance of *Bacillus*, which is in line with the proposition that nematodes can stimulate the quantity and activity of certain bacteria through selective predation or nematode metabolites.^[^ ^69^, ^70^ ^]^ Unlike previous studies that showed that predators reduce N_2_O emissions through broadly preying on microbial communities,^[^ ^6^ ^]^ our study highlights that selective predation and the metabolites supplied by *Protorhabditis* can specifically increase bacterial abundance, particularly of species such as *Bacillus* capable of reducing N_2_O emission. These contrasting effects underline that predator‐mediated changes in microbial communities are not universally inhibitory but are influenced by predator selectivity, feeding mechanisms, and the specific microbial taxa involved. Our findings further support the previous notion that bacterivorous nematodes prefer to feed on Gram‐negative bacteria rather than Gram‐positive bacteria, likely because their thinner cell walls are easier to be digested,^[^ ^71^ ^]^ thereby favoring strains such as *Bacillus* that strongly reduce N_2_O emissions by completing the final step of denitrification. Selective feeding of bacterivorous nematodes on certain bacteria has been shown to be based on olfactory cues,^[^ ^72^ ^]^ but also physical and chemical defense mechanisms of bacteria.^[^ ^73^, ^74^, ^75^ ^]^ By contrast, the impact of soil animal secretions on bacteria remains poorly understood. In our study, we found that betaine, comprising the main compound of metabolites secreted by *Protorhabditis*, stimulates population growth of *Bacillus*. In fact, it has been shown that betaine is needed by many microorganisms including *Bacillus*; it contributes to maintaining osmotic balance and protect cellular functions under stress conditions, such as high salinity or extreme temperatures.^[^ ^76^ ^]^ Moreover, *Bacillus* species may utilize betaine as carbon source,^[^ ^77^, ^78^ ^]^ thereby contributing to *Bacillus* to thrive if resources are limited.^[^ ^79^ ^]^

### 4.2 Bacterial Community Composition and N_2_O Emission

Previous studies focused on direct predation of micro‐predators on microorganisms and its consequence for N_2_O production, but ignored the complexity of interactions in soil micro‐food webs.^[^ ^6^ ^]^ Our results suggest that bacterivore nematodes do not reduce bacterial abundance through predation. Instead, they promote the growth of *Bacillus* species by selectively feeding on competing bacteria and/or producing metabolites. This process enhances the abundance of *nosZ* genes in soil, which in turn contributes to the reduction of N_2_O emissions. *Bacillus* species are among the most common denitrifying bacteria in soil^[^ ^80^ ^]^ and have been shown to reduce N_2_O emission by the expression of N_2_O reductase encoded by the *nosZ* gene.^[^ ^81^, ^82^ ^]^ Previous studies found *Bacillus* to be able to complete the denitrification process with N_2_ as the final gaseous product when using NO_3_^−^‐N as substrate.^[^ ^83^ ^]^ Taken together, the results indicate that increased abundance of certain *Bacillus* species promotes the abundance of the *nosZ* gene reducing N_2_O emission from soils, with these species benefitting from the presence of bacterivorous nematodes such as *Protorhabditis* due to suppressing competitors either via feeding on competitors or via producing growth stimulating substances such as betaine.

**Figure 7:**
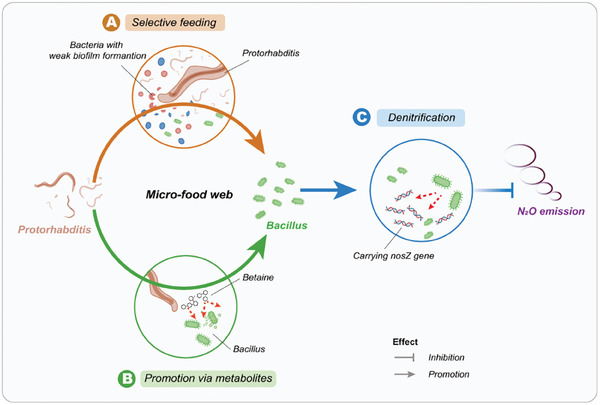
Conceptual diagram illustrating the pathways by which soil bacterivorous nematodes may reduce soil N_2_O emissions.

In conclusion, our study provides novel insight into how bacterivorous nematodes affect bacterial community composition and thereby nitrogen transformation processes by denitrifying bacteria resulting in reduced soil N_2_O emission. We demonstrate that these interactions are based on direct and indirect effects resulting in the facilitation of certain *Bacillus* species carrying *nosZ* genes, with these effects being mediated by *Protorhabditis* selectively feeding on certain bacteria with weak biofilm formation in soil. Betaine exuded by nematodes acts as catalyst promoting *Bacillus* species with high abundance of the *nosZ* gene. The results provide new insights into the mechanistic understanding of how interactions between bacterivorous nematodes and bacteria influence nitrogen transformation processes in soil, thereby affecting soil N_2_O emissions. Our findings contribute to closing knowledge gaps and inform management options aiming at reducing soil N_2_O emissions by manipulating interactions within soil micro‐food webs. However, the results are based on simplified laboratory systems with *Bacillus* and *Protorhabditis* lacking the complex microbial and animal communities in soil. To add realism and come up with land management recommendations, further research is needed to explore the role of interactions between soil nematodes and microorganisms on N_2_O emissions under more natural settings including the full complexity of belowground food webs.^[^ ^84^, ^85^ ^]^ For field‐scale applications, strategies such as controlled nematode inoculation or co‐application with organic fertilizers need to be explored. In addition, challenges such as environmental heterogeneity and nematode survival need to be addressed for successful implementation. To enhance scalability, future studies should investigate the optimal nematode strains, application rates, and their interactions with diverse soil and crop systems under field conditions **Figure** **7**.

## Conflict of Interest

The authors declare no conflict of interest.

## Author Contributions

X.X., X.L.W., S.S.L., R.L., and Q.R.S designed the study. X.X., X.L.W., Y.Y., Y.M., and T.S. performed experiments. X.X. conducted the bioinformatic and statistical analysis. X.X. and X.L.W. prepared the figures and tables. X.X., X.L.W., X.X., S.W.L., V.K. S.S., A.J., S.G., M.H.D., and R.L. wrote the manuscript. All authors edited and approved the final manuscript. X.X., and X.L.W., contributed equally to this work.

## Supporting information



Supporting Information

## Data Availability

The data that support the findings of this study are openly available in [Microbiome to bacterivorous nematodes] at [https://www.ncbi.nlm.nih.gov/sra/PRJNA], reference number [1053638].
