# Supplementary material for: Interactions Between Bacterivorous Nematodes and Bacteria Reduce N2O Emissions
Source: Adv Sci (Weinh). 2025 Jan 31;12(12):2413227. doi: 10.1002/advs.202413227 (PMC11948072; doi:10.1002/advs.202413227)
Supplement: Supplementary file 1 — Supporting Information [file ADVS-12-2413227-s001.docx]

**Supplementary Material**

**Interactions between bacterivorous nematodes and bacteria reduce N_2_O emissions**

Authors: Xu Xu^[[1]](#footnote-1),2†^, Xinling Wang^1†^, Ting Sun^1^, Shanshan Liu^1,2^, Menghui Dong^3^, Yang Yue^1^, Yi Min^1^, Alexandre Jousset^1^, Xian Xiao^4^, Shuwei Liu^1,5^, Stefan Geisen^6^, Valentyna Krashevska^7,8^, Qirong Shen^1^, Stefan Scheu^7,9^, Rong Li^1*^

Affiliation:

1. The Sanya Institute of the Nanjing Agricultural University, Key Lab of Organic-Based Fertilizers of China, Jiangsu Provincial Key Lab for Solid Organic Waste Utilization, Jiangsu Collaborative Innovation Center of Solid Organic Wastes, Educational Ministry Engineering Center of Resource-Saving Fertilizers, Nanjing Agricultural University, Nanjing 210095 Jiangsu, China

2. Ecology and Biodiversity Group, Institute of Environmental Biology, Department of Biology, Utrecht University, Padualaan 8, 3584 CH Utrecht, The Netherlands

3. Department of Agroecology, Faculty of Technical Sciences, Aarhus University, Forsøgsvej 1, 4200, Slagelse, Denmark

4. School of Environmental and Safety Engineering, Changzhou University, Changzhou, China

5. Jiangsu Key Laboratory of Low Carbon Agriculture and GHGs Mitigation, College of Resources and Environmental Sciences, Nanjing Agricultural University, Nanjing, China

6. Laboratory of Nematology, Wageningen University, 6700 AA Wageningen, The Netherlands

7. JF Blumenbach Institute of Zoology and Anthropology, University of Göttingen, Göttingen, Germany

8. Senckenberg Biodiversity and Climate Research Centre, Functional Environmental Genomics, Senckenberganlage 25, 60325 Frankfurt, Germany

9. Centre of Biodiversity and Sustainable Land Use, Göttingen, Germany

**
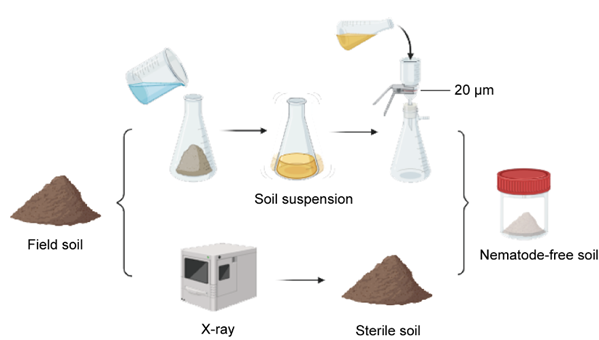
**

**Figure S1. Flowchart for establishing soil without nematodes but with microbial communities.**


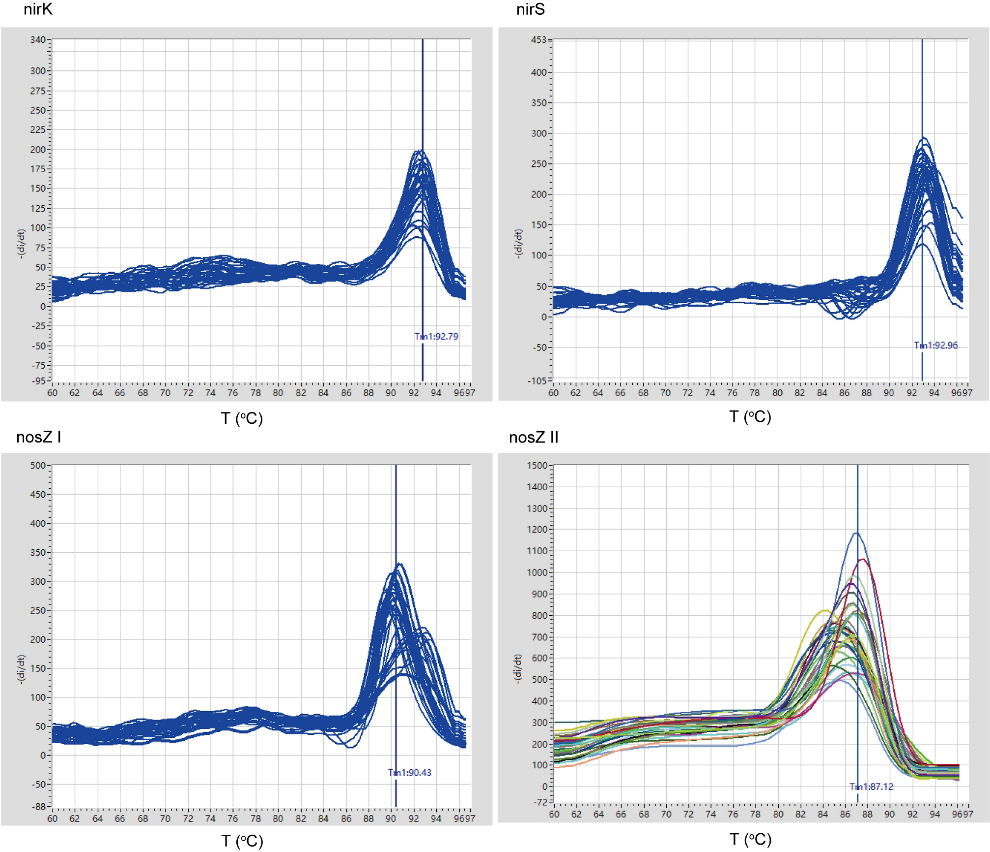


**Figure S2. Melting curve plots of qPCR assays.**


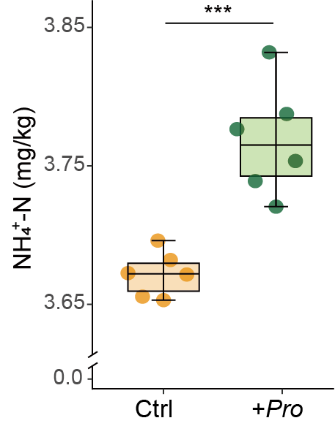


**Figure S3. Soil NH_4_^+^-N content in the control (Ctrl) and with *Protorhabditis* (+*Pro*) treatments (n = 6).** Asterisks, significant difference as indicated by Student’s t-test, ***, *p* < 0.001.


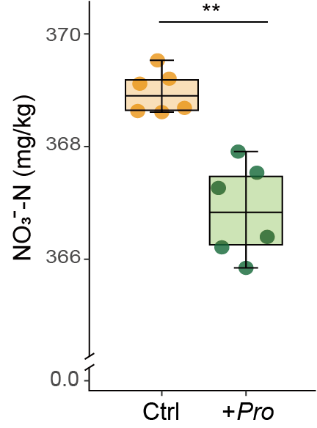


**Figure S4. Soil NO_3_^-^-N content in the control (Ctrl) and with *Protorhabditis* (+*Pro*) treatments (n = 6).** Asterisks, significant difference as indicated by unpaired Student’s t-test, **, *p* < 0.01.


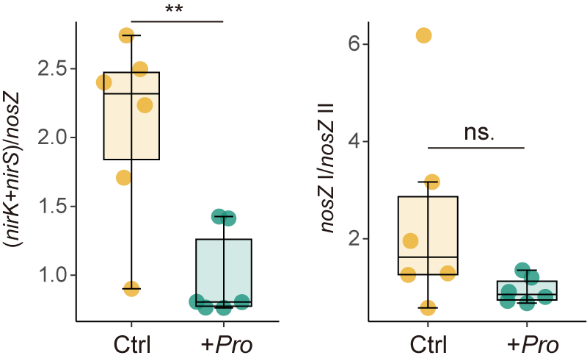


**Figure S5. Ratio of genes in the absence and presence of *Protorhabditis*.** Asterisks, significant differences as indicated by unpaired Student’s t-test (**, p < 0.01; ns, p >0.05.


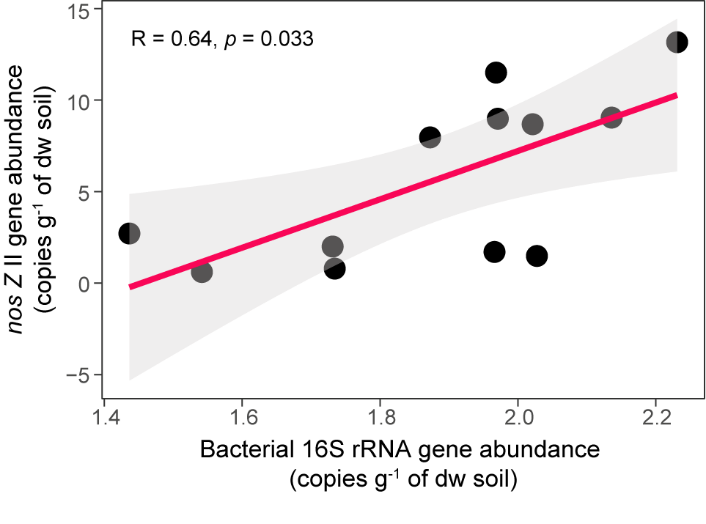


**Figure S6. Spearman correlations between bacterial 16S rDNA gene abundance and *nosZ* II gene abundance.**


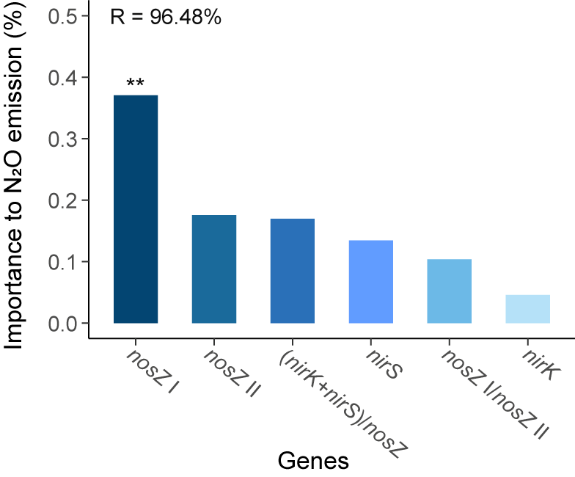


**Figure S7. Contribution of different genes to soil N_2_O emission.**


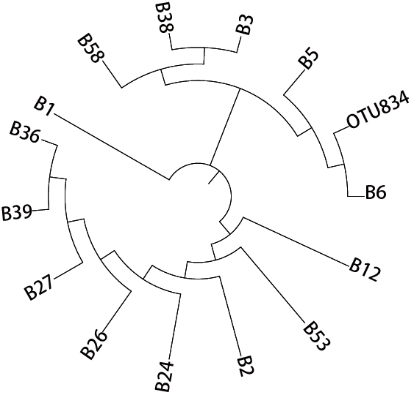


**Figure S8. Identification of *Bacillus* isolates.**


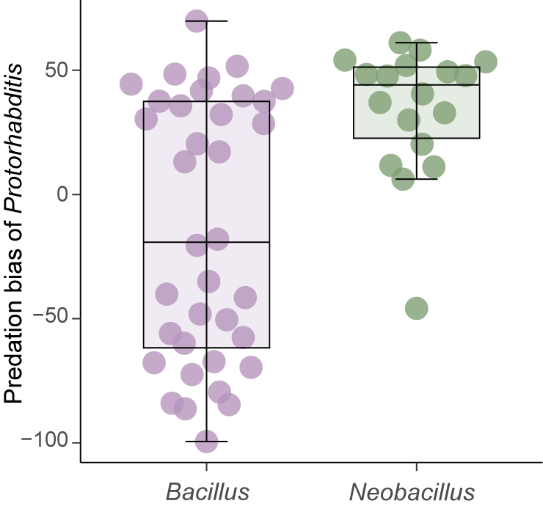


**Figure S9. Response of *Bacillus* strains (B1-B6) and *Neobacillus* strains (N1-N3) to predation by *Protorhabditis.***


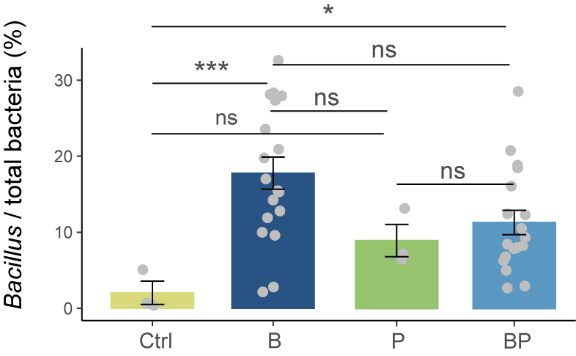


**Figure S10. Proportion of *Bacillus* to total bacteria in soil as indicated by qPCR.** Ctrl, nematode-free soil; B, inoculated with *Bacillus*; P, nematode-free soil inoculated with *Protorhabditis* nematodes; BP, inoculated with *Bacillus* and *Protorhabditis* nematodes.


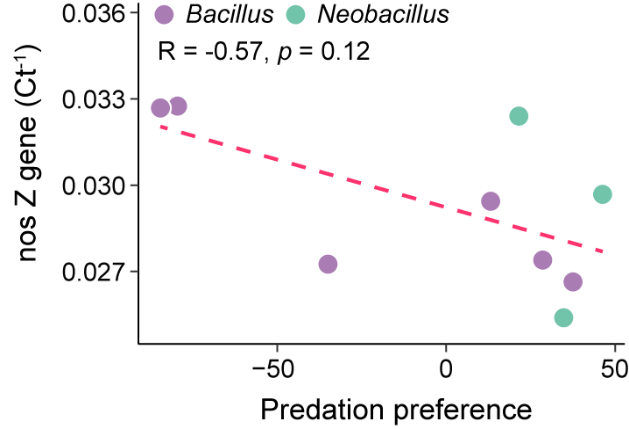


**Figure S11. Correlation between abundance of bacteria carrying *nosZ* gene and predation preference by *Protorhabditis*.**


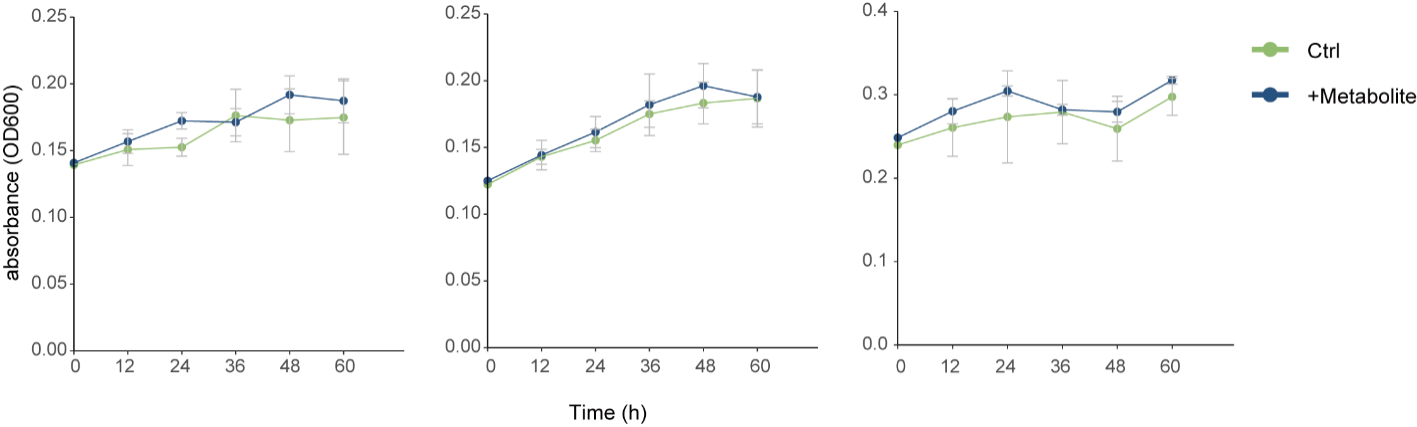


**Figure S12. Growth curve of *Neobacillus* strains (N1-N3) at metabolite of *Protorhabditis.***


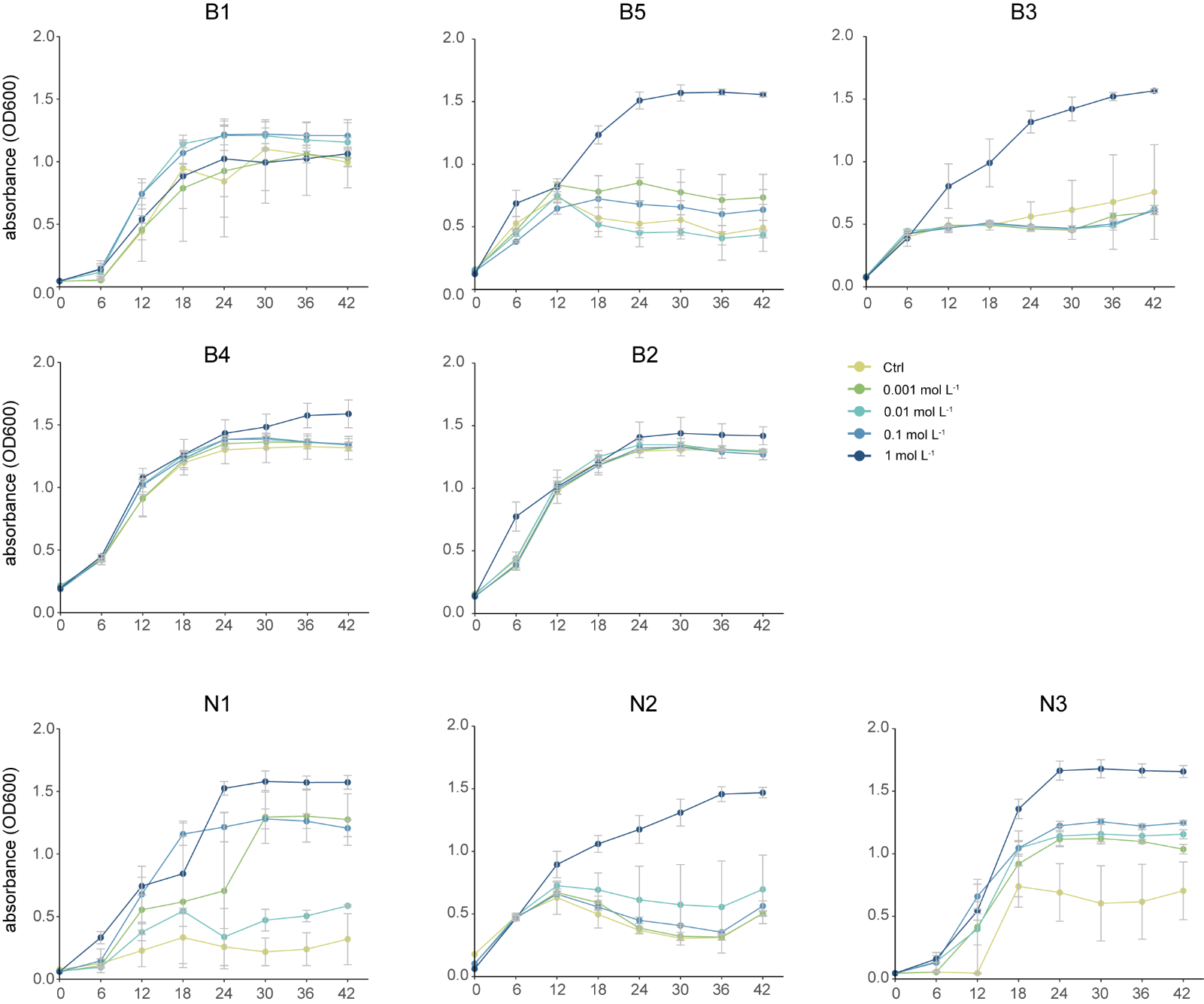


**Figure S13.** **Growth curve of *Bacillus* strains and *Neobacillus* strains at different concentrations of betaine (0.001, 0.01, 0.10, and 1.00 mol L-1).**

**Table S1. Soil properties under different fertilization regimes.** pH, soil pH; SOC, soil organic carbon; BD, bulk density; C/N, soil C/N ratio; TN, total nitrogen; TK, total potassium; AP, available phosphorus; AK, available potassium. Data are means ± SD (n = 3).

|  | **pH** | **SOC**  **(g/kg)** | **BD**  **(g/cm^3^)** | **C/N** | **TN**  **(g/kg)** | **TK**  **(g/kg)** | **AP**  **(mg/kg)** | **AK**  **(mg/kg)** |
| --- | --- | --- | --- | --- | --- | --- | --- | --- |
| **Soil** | 6.7±0.20 | 8.81±1.02 | 1.12±0.09 | 7.01±0.05 | 1.12±0.01 | 6.07±0.91 | 67.82±3.12 | 189±14.00 |

**Table. S2 Information on the PCR program and barcode strategy for each sample.**

| **Target gene** | **Primer** | **Sequence (5′-3′)** | **Thermal profile (qPCR)** |
| --- | --- | --- | --- |
| *nirK* | F1aCu | ATCATGGTSCTGCCGCG | 30 s at 95°C, 44 cycles consisting of 5 s at 95°C, 35 s at 60°C and 30 s at 72°C, 10 s at 95 °C. |
|  | R3Cu | TTGGTGTTRGACTAGCTCCG |  |
| *nirS* | Cd3aF | GTSAACGTSAAGGARACSGG | 30 s at 95°C, 44 cycles consisting of 5 s at 95°C, 45 s at 55°C and 45 s at 72°C, 10 s at 95 °C. |
|  | R3cd | GASTTCGGRTGSGTCTTGA |  |
| clade I *nosZ* | *nosZ*2F | CGCRACGGCAASAAGGTSMSSGT | 30 s at 95°C, 44 cycles consisting of 5 s at 95°C, 35 s at 60°C and 30 s at 72°C, 10 s at 95 °C. |
|  | *nosZ*2R | CAKRTGCAKSGCRTGGCAGAA |  |
| clade II *nosZ* | *nosZ*-II-F | CTIGGICCIYTKCAYAC | 2 min at 95°C, 40 cycles consisting of 15 s at 95°C, 15 s at 55°C and 20 s at 72°C, 5 s at 95 °C. |
|  | *nosZ*-II-R | GCIGARCARAAITCBGTR C |  |
| *nosZ* | *nosZ* 1527F | CGCTGTTCHTCGACAGYCA | 30 s at 95°C, 44 cycles consisting of 5 s at 95°C, 35 s at 60°C and 30 s at 72°C, 10 s at 95 °C. |
|  | *nosZ*1773R | ATRTCGATCARCTGBTCGTT |  |

**Table. S3 The qPCR efficiencies of each gene in denitrification.**

| Gene | qPCR efficiency |
| --- | --- |
| *nosZ* I | 90.24% |
| *nirK* | 85.59% |
| *nirS* | 92.64% |
| *nosZ* II | 90.33% |

**Table. S4 Linear discriminant analysis (LDA) under Ctrl and +*Pro* treatments (n = 6).**

| **OTU** | **Enriched Treatment** | **LDA Score (log 10)** | ***P*** |
| --- | --- | --- | --- |
| OTU1869 | +*Pro* | 2.976535 | 0.003346 |
| OTU251 | +*Pro* | 2.504075 | 0.002093 |
| OTU218 | +*Pro* | 2.651744 | 0.002093 |
| **OTU834** | **+*Pro*** | **3.902413** | **0.003948** |
| OTU4 | +*Pro* | 3.808529 | 0.003885 |
| OTU31 | +*Pro* | 3.318011 | 0.002802 |
| OTU71 | +*Pro* | 3.004155 | 0.003885 |
| OTU50 | +*Pro* | 3.135925 | 0.002093 |
| OTU55 | +*Pro* | 3.052026 | 0.002802 |
| OTU173 | +*Pro* | 2.623036 | 0.003885 |
| OTU82 | +*Pro* | 2.684466 | 0.006485 |
| OTU45 | Control | 2.571331 | 0.019805 |
| OTU2 | Control | 3.176003 | 0.016309 |
| OTU13 | Control | 3.119062 | 0.010406 |

**Table. S5 The *nosZ* gene sequences detected in 6 *Bacillus* isolates (n = 6).**

| **No** | **Ct** | **Anova-*test* (Tukey HSD)** | **Tm** |
| --- | --- | --- | --- |
| B1 | 37.54 ± 0.65 | a | 90.18 ± 1.65 |
| B2 | 32.69 ± 0.57 | c | 90.29 ± 0.30 |
| B3 | 33.97 ± 0.86 | b | 87.41 ± 2.91 |
| B4 | 30.53 ± 0.11 | d | 92.50 ± 0.08 |
| B5 | 36.50 ± 0.41 | a | 89.67 ± 0.43 |
| B6 | 30.60 ± 0.85 | d | 86.67 ± 2.19 |

1. * Correspondence: [lirong@njau.edu.cn](mailto:lirong@njau.edu.cn)

   ^†^ Xu Xu and Xinling Wang contributed equally to this work. [↑](#footnote-ref-1)
